# Incidence, life expectancy and prognostic factors in cancer patients under prolonged mechanical ventilation: a nationwide analysis of 5,138 cases during 1998-2007

**DOI:** 10.1186/cc12823

**Published:** 2013-07-22

**Authors:** Chih-Yuan Shih, Mei-Chuan Hung, Hsin-Ming Lu, Likwang Chen, Sheng-Jean Huang, Jung-Der Wang

**Affiliations:** 1 Department of Family Medicine, National Taiwan University Hospital Jin-Shan Branch, No.51, Nanshih, Jinshan District, New Taipei City 208, Taiwan; 2 Department of Public Health, National Cheng Kung University College of Medicine, No.1, University Road, Tainan 701, Taiwan; 3 Institute of Population Health Sciences, National Health Research Institutes, 35 Keyan Road, Zhunan 350, Taiwan; 4 Institute of Public Health, School of Medicine, National Yang-Ming University, No.155, Sec.2, Linong Street, Taipei 112, Taiwan; 5 National Taiwan University Hospital Jin-Shan Branch, No.51, Nanshih, Jinshan District, New Taipei City 208, Taiwan; 6 Department of surgery, College of Medicine, National Taiwan University, No.1, Sec.1, Jen-Ai Road , Taipei 100, Taiwan; 7 Departments of Internal Medicine and Occupational and Environmental Medicine, National Cheng Kung University Hospital, No.138, Sheng-Li Road, Tainan 704, Taiwan

## Abstract

**Introduction:**

This study is aimed at determining the incidence, survival rate, life expectancy, quality-adjusted life expectancy (QALE) and prognostic factors in patients with cancer in different organ systems undergoing prolonged mechanical ventilation (PMV).

**Methods:**

We used data from the National Health Insurance Research Database of Taiwan from 1998 to 2007 and linked it with the National Mortality Registry to ascertain mortality. Subjects who received PMV, defined as having undergone mechanical ventilation continuously for longer than 21 days, were enrolled. The incidence of cancer patients requiring PMV was calculated, with the exception of patients with multiple cancers. The life expectancies and QALE of patients with different types of cancer were estimated. Quality-of-life data were taken from a sample of 142 patients who received PMV. A multivariable proportional hazards model was constructed to assess the effect of different prognostic factors, including age, gender, type of cancer, metastasis, comorbidities and hospital levels.

**Results:**

Among 9,011 cancer patients receiving mechanical ventilation for more than 7 days, 5,138 undergoing PMV had a median survival of 1.37 months (interquartile range [IQR], 0.50 to 4.57) and a 1-yr survival rate of 14.3% (95% confidence interval [CI], 13.3% to 15.3%). The incidence of PMV was 10.4 per 100 ICU admissions. Head and neck cancer patients seemed to survive the longest. The overall life expectancy was 1.21 years, with estimated QALE ranging from 0.17 to 0.37 quality-adjusted life years for patients with poor and partial cognition, respectively. Cancer of liver (hazard ratio [HR], 1.55; 95% CI, 1.34 to 1.78), lung (HR, 1.45; 95% CI, 1.30 to 1.41) and metastasis (HR, 1.53; 95% CI, 1.42 to 1.65) were found to predict shorter survival independently.

**Conclusions:**

Cancer patients requiring PMV had poor long-term outcomes. Palliative care should be considered early in these patients, especially when metastasis has occurred.

## Introduction

The number of cancer patients has been steadily increasing, and cancer has become the leading cause of death in many countries [1,2]. Patients with malignancies are at risk of developing acute respiratory failure due to the underlying cancer as well as to its treatment, and many of these patients will need mechanical ventilation support while in the ICU. In hospitalized patients with solid tumors, approximately 1% have been diagnosed with acute respiratory failure [3]. The incidence varies from 9% to almost half of the population in patients with hematologic malignancies [3,4]. Studies of patients with cancer admitted to ICUs have demonstrated that mechanical ventilation has been provided for 42% to 83% of these patients [3,5-9].

The number of patients requiring prolonged mechanical ventilation (PMV), defined as having undergone mechanical ventilation continuously for longer than 21 days, is rapidly increasing because of the advances in intensive care [10]. During 2009, approximately 30,000 patients required PMV as recorded by the National Health Insurance (NHI) of Taiwan, which accounted for 4.76% of total NHI healthcare expenditures. This financial burden has now become one of the major threats to the sustainability of the NHI [11,12]. In our previous study, cancer patients requiring PMV account for up to 10.6% of all PMV patients [13]. Although the NHI reimburses all the direct healthcare expenditures of these patients, there is still out-of-pocket money spent for indirect care by the patients' families that results in a financial burden [14]. Moreover, withdrawal of mechanical ventilation was not legally allowed in Taiwan before 2011. Thus, it would help patients and their families to know which patients are least likely to survive on a long-term basis so that further invasive interventions could be reconsidered.

Therefore, accurate prognoses are essential to propose and establish a sustainable national policy and to facilitate communication among different stakeholders. There is limited information on lifetime outcomes in cancer patients who have required PMV. The aims of this study were to determine the incidence and life expectancy of cancer patients requiring PMV and to identify factors associated with lifetime survival, as well as the quality-adjusted life expectancy (QALE) of cancer patients undergoing PMV.

## Materials and methods

### Study population and data sets

**Figure 1:**
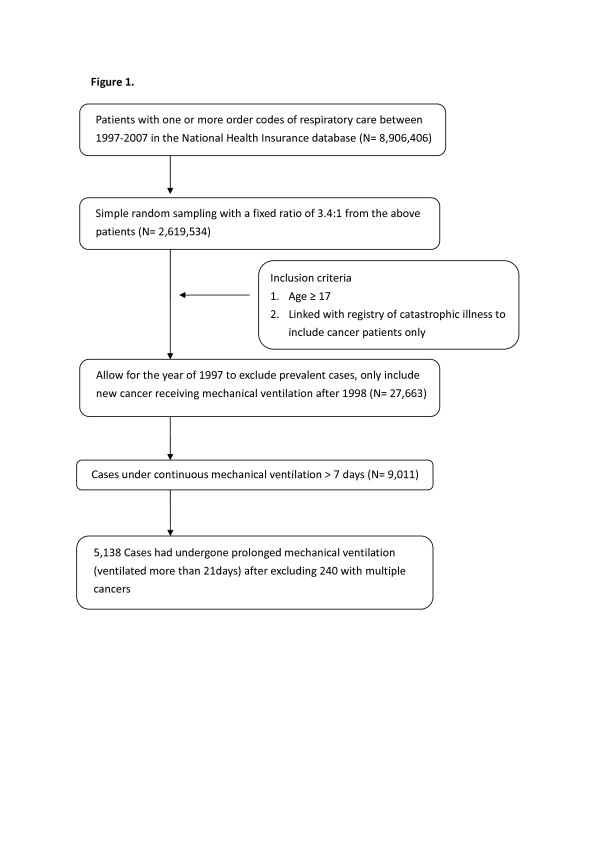
**Flow diagram of selection process used for the study cohort**.

The current study was approved by the Institutional Review Board of National Taiwan University Hospital (IRB 200912072R). We retrieved the data from the National Health Insurance Research Database (NHIRD), a reimbursement data file that we obtained from the NHI of Taiwan. It was transformed into a research database by the National Health Research Institutes (Chunan, Taiwan). The identification numbers of all individuals in the reimbursement data file were encrypted to protect the patients' privacy. These files contained detailed demographic data (including birthdate and gender) and information regarding the healthcare services provided for each patient, including all payments for outpatient visits, hospitalizations, prescriptions and intervention procedures. In addition, up to five diagnoses were provided for the hospitalization. In total, 8,906,406 individuals had undergone invasive or noninvasive respiratory care at least once during the period from 1997 to 2007. This number corresponds to approximately 29.4% of the entire insured population. Because the government has established guidelines stating that no more than 10% of all data can be drawn for research, we applied for a random sample of these patients with a 3.4:1 sampling ratio. Namely, 1 record of a patient who had undergone respiratory care was randomly drawn from every 3.4 records so that the total data would be close to the 10% limit. Subjects who were over the age of 17 years and had undergone extended mechanical ventilation for longer than 7 days were enrolled, wherein PMV was defined as patients requiring more than 21 days of mechanical ventilation [15,16]. To ensure that all of the patients were incident cases, we excluded all prevalent cases found in 1997 and began the collection in 1998. To select cancer patients, we linked our data set with the registry of cancer under NHIRD catastrophic illness and excluded cases with multiple cancer diagnoses, as summarized in Figure 1. The incidences were the risks of PMV among different types of cancer patients admitted to the ICU during a period from 1998 to 2007.

### Statistical analysis

#### Determinants or prognostic factors of survival

Age, gender, hospital level and comorbidities were included as the major determinants with which to explore the survival of these patients. Differences in survival were examined across gender and four categories of age (younger than 65, 65 to 74, 75 to 84, and 85 years and older). The hospital level was retrieved from the NHI claim codes and was classified into district hospital (usually fewer than 250 beds), regional hospital (usually 250 to 1,000 beds) or medical center (usually more than 1,000 beds). The data for each inpatient hospitalization included up to five diagnoses, which were coded according to the International Classification of Diseases, Ninth Revision, Clinical Modification. The diagnoses were reclassified into 260 categories according to the Clinical Classifications Software (CCS) for ICD-9-CM [17]. The classification of cancer types and metastatic status in our study were simplified from the multilevel CCS codes to collect sufficient numbers of subcohorts. Comorbidities were identified from the single-level CCS and reclassified into 40 broad categories excluding cancer. Comorbidities occurring in less than 5% of the PMV study population were excluded. Univariate Cox regression analysis was used first to examine the correlation between each prognostic variable and the length of survival. Spearman's rank correlation was calculated to explore any collinearity between the studied variables. Multivariable Cox regression analyses were performed to determine the prognostic impact of cancer types after adjusting for potential confounding variables. Variables considered in the models included gender, four categories of age, period of receiving PMV treatment (1998 to 2000, 2001 to 2004 and 2005 to 2007), extent of cancer (local vs metastasis), cancer types (organ system or location), acute comorbidities, concurrent organ disorder (chronic comorbidities) and hospital level (district hospital, regional hospital or medical center). Stepwise selection processes were applied to select the comorbidities of prognostic relevance. Acute and chronic comorbidities were considered both separately and concurrently in the Cox regression models. All statistical analyses were performed by using SAS version 9.1 software (SAS Institute, Cary, NC, USA).

#### Survival analysis and estimation of life expectancy

**Figure 2:**
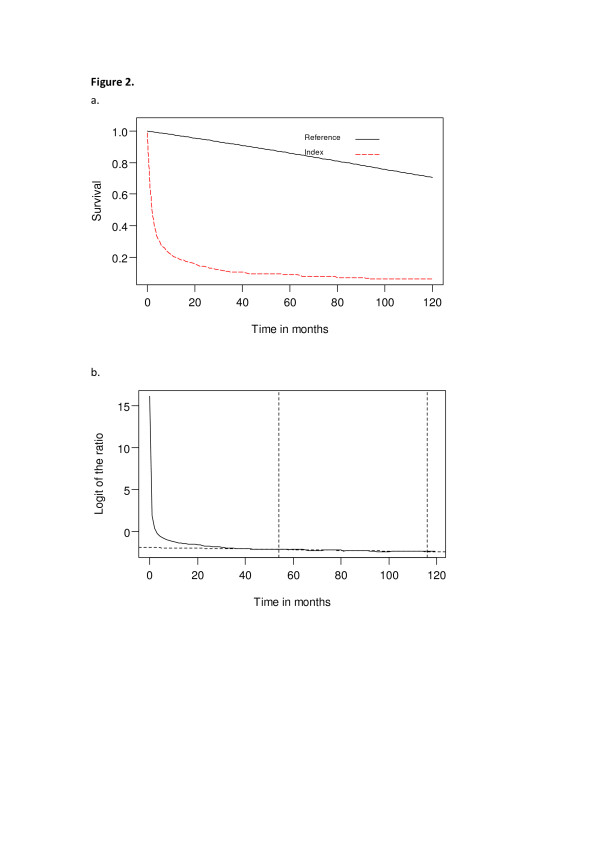
**Survival analysis and estimation of life expectancy**. **(A) **Estimated survival curve for patients with head and neck cancer undergoing prolonged mechanical ventilation (indicated by dashed line) and age- and gender-matched referents generated by Monte Carlo method based on life tables of vital statistics of Taiwan. **(B) **Plot of logit-transformed curve of the survival ratio between index and referents populations showing the fulfillment of constant excess hazard.

Each new patient who fulfilled the definition of mechanical ventilation was followed from the seventh day of mechanical ventilation until he or she died or was censored on 31 December, 2007. The median survival and long-term survival rates were estimated using the Kaplan-Meier method. Patients who survived more than 21 days were followed under an integrated system of reduced intensive respiratory care [18]. Their lifetime survival was estimated up to 300 months using a linear extrapolation of a logit-transformed curve for the survival ratio between the PMV and an age- and gender-matched reference population generated by the Monte Carlo method from the life table of the general population of Taiwan (Figure 2). The detailed method and mathematical proof assuming a constant excess hazard were described in our previous reports [19-21]. To facilitate computation, we used iSQoL, a software program that was built on the R programming language for lifetime expectancy estimation and 300-month extrapolation that can be downloaded for free from the website [22].

#### Estimation of quality-adjusted life expectancy

The lifetime survival probabilities along the duration to dates (or time after PMV onset) were multiplied (or adjusted) with the quality-of-life (QoL) values to obtain a quality-adjusted survival curve. The sum of the total area under this curve was the QALE with quality-adjusted life years (QALYs) as the common unit [23]. Our QoL data were taken from a sample of 142 patients under PMV and measured using the EQ-5D Health Questionnaire and were classified into either partial or poor cognition [24,25]. Partial cognition was defined as an Mini Mental State Examination score higher than 15. Poor cognition was categorized as scores equal to or less than 15. The detailed estimation method was summarized and reported in a previous article evaluating the cost-effectiveness of PMV treatment [14].

## Results

### Patient characteristics

A total of 9,011 cancer patients under extended mechanical ventilation were included during the study period. The median survival was generally less than 2 months, and the 1-yr survival rate was mostly less than 20%. Among those patients studied, 5,138 had undergone mechanical ventilation for more than 21 days. Their mean age was 69.1 years (SD ± 13.8), and 33% were females. The median survival was 1.37 months (interquartile range [IQR], 0.50 to 4.57) (Table 1), which is slightly longer than the 1.13 months (IQR, 0.47 to 4.30) of the 240 excluded cases with multiple cancers. About one-third of the cases were patients with cancers occurring in the head and neck or in the respiratory tract. About one-fourth were metastasized cancers, indicating that advanced cancer patients treated with PMV were not uncommon. The most frequently encountered acute comorbidities were septicemia (22.4%), followed by gastrointestinal hemorrhage (10.9%) and shock (10.6%).

**Table 1:** Characteristics and survival of cancer patients categorized by duration of mechanical ventilation (1998 to 2007).^a^

	Duration of mechanical ventilation						
	
	>7 days (extended MV)			>21 days (prolonged MV)			
Variables	*N*	Median survival, months (IQR)	1-yr survival rate, % (95% CI)	*N*	Median survival, months (IQR)	1-yr survival rate, % (95% CI)	Life expectancy, yr (SE)
Total	9,011	1.54 (0.67 to 5.90)	17.6 (16.8 to 18.4)	5138	1.37 (0.50 to 4.57)	14.3 (13.3 to 15.3)	1.21 (0.10)
Gender and age (yr)							
Male							
17 to 64	2,282	1.64 (0.66 to 8.29)	20.4 (18.8 to 22.2)	1,010	1.17 (0.43 to 3.73)	13.9 (11.8 to 16.2)	1.52 (0.22)
65 to 74	1,686	1.50 (0.67 to 5.10)	16.7 (14.9 to 18.5)	986	1.37 (0.50 to 4.23)	12.8 (10.8 to 15.1)	1.02 (0.13)
75 to 84	1,729	1.50 (0.70 to 4.94)	15.1 (13.4 to 16.9)	1,116	1.40 (0.53 to 4.63)	13.3 (11.3 to 15.4)	0.86 (0.09)
85 or older	425	1.64 (0.77 to 4.50)	14.9 (11.6 to 18.6)	318	1.80 (0.63 to 4.73)	16.4 (12.4 to 20.9)	0.95 (0.19)
Female							
17 to 64	1,129	1.40 (0.60 to 6.84)	19.8 (17.5 to 22.3)	573	1.30 (0.43 to 5.43)	17.2 (14.2 to 20.5)	1.49 (0.31)
65 to 74	788	1.54 (0.64 to 5.27)	17.2 (14.6 to 20.0)	459	1.40 (0.63 to 4.53)	13.9 (10.9 to 17.3)	1.03 (0.24)
75 to 84	727	1.50 (0.67 to 5.50)	14.3 (11.8 to 17.1)	499	1.47 (0.57 to 5.83)	14.8 (11.7 to 18.2)	1.07 (0.30)
85 or older	234	1.84 (0.86 to 6.67)	17.6 (12.9 to 23.0)	177	1.70 (0.73 to 6.20)	16.4 (11.1 to 22.6)	0.82 (0.24)
Extent of cancer							
Local	5,954	1.87 (0.79 to 8.32)	20.6 (19.5 to 21.7)	3,900	1.60 (0.57 to 5.97)	16.9 (15.7 to 18.2)	1.36 (0.11)
Metastasis	3,057	1.07 (0.50 to 3.12)	11.7 (10.6 to 12.9)	1,238	0.93 (0.37 to 2.10)	5.9 (4.6 to 7.3)	0.64 (0.15)
Cancer type							
Head and neck	1,432	2.60 (0.89 to 13.32)	26.0 (23.7 to 28.3)	705	1.77 (0.70 to 7.57)	20.0 (17.0 to 23.1)	1.57 (0.45)
Bone and connective tissue	52	1.37 (0.77 to 2.74)	10.0 (3.7 to 20.1)	38	0.87 (0.37 to 1.77)	2.8 (2.1 to 12.2)	-^b^
Liver	775	0.87 (0.40 to 2.50)	10.5 (8.4 to 12.8)	297	1.00 (0.40 to 2.43)	6.8 (4.2 to 10.2)	0.45 (0.19)
Lung	1,752	1.10 (0.56 to 2.93)	10.5 (9.1 to 12.1)	965	1.00 (0.37 to 2.57)	6.6 (5.1 to 8.3)	0.77 (0.13)
Esophagus	495	2.07 (0.86 to 9.17)	22.5 (18.8 to 26.3)	232	1.03 (0.47 to 2.83)	10.2 (6.6 to 14.6)	0.98 (0.33)
Lymphatic and hematopoietic tissue	578	1.20 (0.57 to 4.47)	14.2 (11.5 to 17.2)	289	1.27 (0.43 to 4.33)	14.3 (10.4 to 18.7)	1.12 (0.36)
Urinary organs	420	1.74 (0.90 to 4.94)	16.0 (12.6 to 19.8)	306	1.30 (0.60 to 3.87)	12.8 (9.2 to 16.9)	0.88 (0.22)
Female genital	345	1.37 (0.67 to 5.37)	17.2 (13.4 to 21.5)	214	1.23 (0.50 to 5.23)	14.1 (9.8 to 19.3)	0.98 (0.63)
Pancreas and other GI organs	176	1.27 (0.61 to 3.57)	13.2 (8.6 to 18.7)	99	1.33 (0.43 to 4.90)	15.1 (8.9 to 22.9)	-^b^
Stomach	503	1.57 (0.74 to 5.44)	17.7 (14.4 to 21.3)	305	1.60 (0.60 to 5.60)	17.0 (12.9 to 21.6)	0.98 (0.38)
Thyroid	77	1.81 (0.87 to 10.34)	24.9 (15.7 to 35.2)	56	1.27 (0.50 to 5.17)	18.6 (9.5 to 30.0)	-^b^
Breast	375	1.40 (0.57 to 4.97)	17.1 (13.3 to 21.3)	223	1.40 (0.60 to 6.47)	18.9 (13.8 to 24.6)	1.17 (0.48)
Skin	100	2.30 (0.97 to 9.10)	21.3 (13.7 to 30.0)	83	1.83 (0.60 to 6.33)	18.1 (10.6 to 27.3)	-^b^
Brain and nervous system	280	2.40 (1.17 to 7.60)	18.0 (13.5 to 23.0)	228	1.97 (0.83 to 7.13)	15.8 (11.2 to 21.2)	1.18 (0.50)
Colorectal	1,019	1.91 (0.80 to 9.60)	22.8 (20.2 to 25.5)	650	1.77 (0.60 to 7.07)	18.8 (15.8 to 22.0)	1.47 (0.23)
Male genital	435	1.81 (0.77 to 7.14)	18.6 (14.9 to 22.5)	303	2.17 (0.70 to 9.20)	19.9 (15.4 to 24.8)	1.15 (0.41)
Others	197	1.47 (0.77 to 7.30)	20.8 (15.3 to 26.7)	145	1.03 (0.40 to 3.70)	14.9 (9.5 to 21.3)	-^b^
Acute comorbidities							
Acute renal failure	630	0.90 (0.50 to 1.80)	7.4 (5.4 to 9.8)	359	0.90 (0.37 to 1.93)	8.4 (5.6 to 11.9)	0.44 (0.20)
Septicemia	2,013	1.02 (0.56 to 2.40)	9.8 (8.5 to 11.2)	1,155	0.93 (0.37 to 2.60)	10.2 (8.5 to 12.2)	0.94 (0.24)
Shock	1,038	0.89 (0.44 to 2.07)	9.0 (7.3 to 10.8)	547	0.87 (0.33 to 2.40)	9.4 (7.0 to 12.1)	0.96 (0.20)
GI hemorrhage	941	1.40 (0.60 to 4.70)	15.0 (12.8 to 17.4)	559	1.20 (0.40 to 4.27)	14.2 (11.4 to 17.3)	1.12 (0.26)
Other injury and poisoning	729	2.90 (1.00 to 16.57)	28.4 (25.1 to 31.8)	392	2.07 (0.77 to 8.50)	21.8 (17.7 to 26.1)	1.61 (0.45)
Cerebrovascular disease	592	2.37 (0.89 to 12.14)	25.2 (21.7 to 28.9)	426	2.67 (0.83 to 12.23)	25.0 (20.8 to 29.4)	1.61 (0.38)
Concurrent organ disorder (chronic comorbidities)							
Hypertension	615	2.60 (0.89 to 16.04)	27.3 (23.7 to 31.0)	354	2.20 (0.80 to 8.90)	21.7 (17.3 to 26.4)	1.83 (0.44)
COPD	615	2.10 (0.87 to 9.97)	23.0 (19.6 to 26.4)	428	2.00 (0.67 to 8.73)	20.6 (16.8 to 24.6)	1.34 (0.38)
Hospital level							
District	977	1.40 (0.63 to 4.04)	12.6 (10.5 to 14.8)	694	1.40 (0.57 to 5.03)	14.3 (11.7 to 17.1)	1.17 (0.31)
Regional	3,058	1.44 (0.64 to 4.57)	14.4 (13.2 to 15.8)	1,772	1.27 (0.50 to 3.87)	12.2 (10.7 to 13.8)	1.04 (0.22)
Medical center	4,976	1.67 (0.70 to 8.02)	20.5 (19.3 to 21.6)	2,672	1.40 (0.53 to 5.07)	15.6 (14.2 to 17.1)	1.37 (0.18)

^a^CI, confidence interval; COPD, chronic obstructive pulmonary disease; GI, gastrointestinal; IQR, interquartile range; MV, mechanical ventilation; SE, standard error of the mean. ^b^Life expectancy could not be calculated because of violation of the assumption of constant excess hazard or small numbers.

### Incidence of prolonged mechanical ventilation

The overall incidence of cancer patients requiring PMV was 10.4 per 100 ICU admissions. The incidences of PMV in different types of cancer are summarized in Table 2. Lung cancer patients yielded the highest incidence of PMV (14.9 per 100 ICU admissions). Patients with liver cancer exhibited the lowest incidence of PMV (4.3 per 100 ICU admissions).

**Table 2:** Incidence and the estimated quality-adjusted life expectancy for cancer patients undergone prolonged mechanical ventilation^a^

Type of cancer	Number of ICU admissions	Incidence of PMV per 100 ICU admissions (95% CI)	QALE in QALY units (SE)	
			
			Partial cognition	Poor cognition
Lung	6,114	14.9 (8.4 to 23.5)	0.23 (0.06)	0.11 (0.03)
Others	608	14.3 (8.4 to 23.5)	-^b^	-^b^
Male genital	2,197	14.2 (8.4 to 23.5)	0.33 (0.14)	0.15 (0.06)
Brain & nervous system	1,490	14.0 (7.7 to 24.5)	0.35 (0.19)	0.16 (0.08)
Lymphatic and hematopoietic tissue	2,242	13.3 (7.7 to 22.2)	0.35 (0.16)	0.16 (0.06)
Esophagus	1,781	13.0 (6.9 to 22.2)	0.30 (0.13)	0.14 (0.07)
Skin	579	13.1 (7.7 to 22.2)	-^b^	-^b^
Thyroid	432	12.3 (6.9 to 21.0)	-^b^	-^b^
Stomach	2,647	11.1 (6.2 to 19.7)	0.28 (0.13)	0.13 (0.04)
Colorectal	6,213	10.6 (5.5 to 18.4)	0.45 (0.11)	0.20 (0.06)
Urinary organs	2,883	10.4 (5.5 to 18.4)	0.26 (0.08)	0.12 (0.04)
Bone and connective tissue	339	10.3 (5.5 to 18.4)	-^b^	-^b^
Breast	2,072	9.9 (4.8 to 17.1)	0.34 (0.17)	0.16 (0.06)
Head and neck	7,074	8.8 (4.1 to 15.8)	0.48 (0.21)	0.22 (0.08)
Female genital	2,342	8.5 (4.1 to 15.8)	0.28 (0.23)	0.12 (0.11)
Pancreas and other GI organs	1,097	8.0 (3.5 to 15.8)	-^b^	-^b^
Liver	6,625	4.3 (1.6 to 10.2)	0.12 (0.07)	0.06 (0.02)

^a^CI, confidence interval; GI, gastrointestinal; ICU, intensive care unit; QALY, quality-adjusted life year; SE, standard error of the mean. ^b^QALE could not be calculated because of violation of assumption or small numbers.

### Survival rate, life expectancy and quality-adjusted life expectancy

The results of survival rate, life expectancy and QALE of the study populations categorized using different variables are summarized in Tables 1 and 2. Patients with metastatic cancer status have a generally poor prognosis, with a 1-yr survival rate of 5.9% (95% confidence interval [CI], 4.6% to 7.3%) and estimated life expectancy of 0.64 year. In addition, the life expectancies and QALEs are generally better in patients with head and neck or colorectal cancer. Those patients with liver or lung cancer had a poor prognosis.

### Prognostic factors

The results of three multivariate Cox regression models are summarized in Table 3. Male gender, metastatic cancer and comorbidities of acute renal failure, shock, septicemia and gastrointestinal hemorrhage significantly predicted shorter survival after controlling for other risk factors. Patients treated in medical centers seem to have a slightly better prognosis after adjustment for other predictors (Table 3). In addition, age and period of receiving PMV treatment had no significant prognostic impact on survival for cancer patients.

**Table 3:** Independent risk factors for mortality in cancer patients undergone prolonged mechanical ventilation^a^

Variables	Risk of death	
	
	Crude HR (95% CI)	**Adjusted HR**^b^**(95% CI)**
Gender		
Female	1.00	1.00
Male	1.07 (1.01 to 1.14)	1.12 (1.04 to 1.20)
Age (yr)		
17 to 64	1.00	1.00
65 to 74	0.99 (0.92 to 1.07)	0.99 (0.91 to 1.07)
75 to 84	0.96 (0.89 to 1.03)	1.02 (0.94 to 1.10)
85 or older	0.90 (0.81 to 1.01)	0.98 (0.87 to 1.10)
Extent of cancer		
Local	1.00	1.00
Metastatic	1.56 (1.45 to 1.66)	1.53 (1.42 to 1.65)
Cancer type		
Head and neck	1.00	1.00
Bone and connective tissue	2.08 (1.49 to 2.90)	2.06 (1.48 to 2.88)
Liver	1.65 (1.43 to 1.90)	1.55 (1.34 to 1.78)
Lung	1.58 (1.42 to 1.75)	1.45 (1.30 to 1.61)
Esophagus	1.44 (1.23 to 1.69)	1.32 (1.13 to 1.54)
Lymphatic and hematopoietic tissue	1.26 (1.09 to 1.45)	1.32 (1.14 to 1.52)
Urinary organs	1.23 (1.07 to 1.43)	1.24 (1.07 to 1.43)
Female genital	1.19 (1.01 to 1.40)	1.23 (1.03 to 1.46)
Pancreas and other GI organs	1.19 (0.96 to 1.48)	1.17 (0.94 to 1.47)
Thyroid	1.14 (0.86 to 1.52)	1.17 (0.88 to 1.56)
Breast	1.10 (0.94 to 1.29)	1.14 (0.96 to 1.35)
Skin	1.07 (0.84 to 1.36)	1.08 (0.84 to 1.38)
Stomach	1.09 (0.9 to 1.26)	1.08 (0.93 to 1.25)
Brain and nervous system	1.01 (0.8 to 1.19)	1.04 (0.89 to 1.22)
Colorectal	1.05 (0.93 to 1.17)	1.04 (0.92 to 1.17)
Male genital	0.95 (0.82 to 1.10)	0.93 (0.80 to 1.09)
Others	1.28 (1.06 to 1.54)	1.14 (0.95 to 1.39)
Acute comorbidities^c^		
Acute renal failure	1.43 (1.28 to 1.60)	1.46(1.30 to 1.64)
Septicemia	1.33 (1.24 to 1.43)	1.24(1.15 to 1.34)
Shock	1.32 (1.21 to 1.45)	1.22(1.10 to 1.34)
GI hemorrhage	1.07 (0.98 to 1.18)	1.10(1.01 to 1.21)
Other injury and poisoning	0.78 (0.70 to 0.87)	0.83 (0.74 to 0.92)
Cerebrovascular disease	0.69 (0.62 to 0.77)	0.78 (0.69 to 0.87)
Concurrent organ disorder (chronic comorbidities) ^C^		
Hypertension	0.74 (0.65 to 0.83)	0.82 (0.73 to 0.93)
COPD	0.81 (0.73 to 0.91)	0.88 (0.79 to 0.98)
Hospital level		
District	1.00	1.00
Regional	1.12 (1.02 to 1.23)	1.02 (0.93 to 1.12)
Medical center	1.00 (0.91 to 1.09)	0.91 (0.83 to 1.00)

^a^CI, confidence interval; COPD, chronic obstructive pulmonary disease; GI, gastrointestinal. ^b^Hazard ratio adjusted for cancer type, gender, age, extent of cancer, acute and chronic comorbidities, and hospital level. ^c^The reference group is without the comorbidity listed below.

## Discussion

Although there are studies reporting the poor prognosis of patients with advanced cancer hospitalized in the ICU and under mechanical ventilation, to the best of our knowledge, our study is the first to provide the crucial estimates of survival rates, life expectancies and QALE in these patients categorized by metastatic status and types of cancer based on a national database, as summarized in Tables 1 and 2. We found the median survival of cancer patients under more than 7 days of mechanical ventilation is generally less than 2 months, with the exception of those with cancer of the head, neck, esophagus, skin or nervous system. Half of those patients under PMV survived less than 1.4 months with an overall 1-yr survival rate of 14.3% (95% CI, 13.3% to 15.3%), which is generally poorer than that of patients with other comorbidities [13].

Among different types of cancer, those occurring in the lung and nervous system seemed to have the highest incidence of ICU admissions (Table 2). This might be associated with direct tumor involvement of the respiratory tract and/or the control of ventilation. Patients with liver cancer were found to exhibit the lowest incidence of PMV, which may be related to their lower survival rate and life expectancy (Table 1).

Previous studies of cancer patients requiring ventilator support in the ICU could differentiate cancer types into only solid and hematological malignancy because of small numbers [7,26,27]. Among them, Soares *et al*. reported a 60% rate of 6-month mortality for 163 patients who had a prolonged ICU length of stay (21 days or longer), and the number of organ failures, old age and poor performance status were significantly associated with poor outcomes [27]. Nonetheless, in our study, with 10-yr follow-up of a nationwide collection of cases, we have been able to detect cancer occurring in different organ systems as a predictor of lifetime mortality after adjustment for multiple confounders (Table 3). It is also noteworthy that age is not related to the outcomes in our study, which might indicate that cancer patients under PMV are usually at the most severe stage, with a life expectancy of 1.21 yr. Thus, age did not appear to influence such an overall poor prognosis.

Critical care with mechanical ventilation has been advocated as a therapeutic trial for critically ill cancer patients, but failure of such care might signal a need for a transition from curative to palliative care [28,29]. Patients and their families may be more likely to accept revision of the goal of care when there is little hope for meaningful recovery [30]. In many occasions, however, stakeholders have too little important prognostic information, especially with regard to reliable estimates of life expectancies for different conditions, to use in making decisions [31]. The long-term outcomes and prognostic factors for PMV cancer patients in our study may thus be useful for communication among all stakeholders to facilitate patient-centered clinical decisions and possible early integration of palliative care, which is very crucial in Taiwan, where regulations for extubation are rather restrictive. This used to require the signatures of all first-degree relatives; but, in 2013, the rule was changed to require only one signature.

In addition to survival, a more ideal outcome evaluation must also take QoL into consideration. Assuming that the QoL of these patients is similar to that of other PMV patients, we tried to categorize them into patients with poor cognition and those with partial cognitive ability. Using that method, the overall QALE would be about 0.17 to 0.37 QALY. Even for patients with colorectal cancer requiring PMV who had the longest survival, the QALE was only 0.20 to 0.45 QALYs for patients with poor cognition and those with partial cognitive ability, respectively. Further evaluation of the cost per QALY in another study for patients under PMV found that cancer patients spent $64,708 and US$148,829 per QALY with partial versus poor cognition, respectively. Both figures cost more than three times Taiwan's gross domestic product and were not considered cost-effective based on criteria suggested by the World Health Organization's CHOosing Interventions that are Cost-Effective (WHO-CHOICE) guideline [32]. The out-of-pocket expenses were estimated to be about one-third those including such spending [14]. Thus, we recommend that palliative care be considered early, especially among patients with metastasis, to avoid a prolonged dying process.

Our study has the following limitations. First, the database did not contain any information regarding the parameters of prior performance status, which has been reported to be predictive for long-term survival in critically ill cancer patients, including those requiring mechanical ventilation [9,33,34]. As poor performance status was usually associated with advanced or metastatic cancer [35], the HR we obtained in this study might be overestimated. Second, because the billing records in the NHIRD provide neither the exact date of tracheostomy nor the main reasons for ICU admission, we were unable to stratify these patients by the stability of their clinical course. Further prospective study is warranted to clarify this issue. Third, decisions to limit therapy, and particularly "do not resuscitate" (DNR) orders, were not recorded in the NHI data. Patients with DNR orders may prevent the use of aggressive treatment, which may be a crucial confounding factor in the survival analysis. Although we recommend that future studies take this factor into account, the magnitude of potential bias resulting from this factor might be quite limited in our present study because all cancer patients under PMV were already intubated, and, because of the legal restrictions in Taiwan before 2011, no one, even with the consent of all family members, was allowed to withdraw PMV. Thus, considerations for these patients were presumed to be those without DNR consent. However, the adaptation of PMV incidence in our study should be cautious because life-supporting treatment would be allowed to be withdrawn in other countries under certain circumstances [36,37].

## Conclusions

Cancer patients undergoing mechanical ventilation for more than 7 days usually had a poor long-term outcome, especially those under PMV, which we defined as continuous mechanical ventilation for more than 21 days. In our opinion, palliative care should be considered early in cancer patients with metastases. The extent of treatment and end-of-life issues should be openly discussed in the early stages of care for cancer patients. In Taiwan, too often this discussion begins after a patient's loss of decision-making ability, suggesting general ignorance of the principle of respect for autonomy. Our results also call to attention the cost-effectiveness of current policies in the care of cancer patients undergoing PMV.

## Key messages

• Half of cancer patients under PMV survived less than 1.4 months, and the overall 1-yr survival rate was 14.3%.

• Cancer of the liver and lung and metastasis independently predict shorter survival.

• We recommend that palliative care be considered early in mechanically ventilated patients with metastatic cancer.

## Abbreviations

CCS: Clinical Classifications Software; NHI: National Health Insurance; PMV: prolonged mechanical ventilation; QALE: quality-adjusted life expectancy.

## Competing interests

The authors declare that they have no competing interests.

## Authors' contributions

CYS performed the analysis and interpretation of data and drafted and revised the manuscript. MCH was involved in the acquisition of data, data analysis, interpretation of data and revision of the manuscript. HML was involved in the acquisition of data and data analysis. LC contributed to the study design, acquisition of data, interpretation of data and revision of the manuscript. SJH contributed to the interpretation of data and revision of the manuscript. JDW contributed to the study concept and design, interpretation of data, and drafting and revision of the manuscript. All authors approved the final version of the manuscript submitted for publication.
